# Genome-wide identification of AP2/EREBP in *Fragaria vesca* and expression pattern analysis of the FvDREB subfamily under drought stress

**DOI:** 10.1186/s12870-021-03095-2

**Published:** 2021-06-26

**Authors:** Chao Dong, Yue Xi, Xinlu Chen, Zong-Ming Cheng

**Affiliations:** 1 College of Horticulture, Nanjing Agricultural University, Nanjing, 210095 Jiangsu China; 2 Shanghai Key Laboratory of Protected Horticultural Technology, Forestry and Fruit Tree Research Institute, Shanghai Academy of Agricultural Sciences (SAAS), Shanghai, 201403 China; 3 Shanghai Center for Plant Stress Biology (PSC), Chinese Academy of Sciences, Shanghai, 201602 China; 4 Department of Plant Sciences, University of Tennessee, Knoxville, TN 37996 USA

**Keywords:** DREB, Structural characteristics, Duplication, Drought stress, *Fragaria vesca*, Expression

## Abstract

**Background:**

Drought is a common phenomenon worldwide. It is also one of the main abiotic factors that affect the growth and quality of strawberry. The dehydration-responsive element binding protein (DREB) members that belong to the APETALA2/ethylene-responsive element binding protein (AP2/EREBP) superfamily are unique transcription factors in plants that play important roles in the abiotic stress response.

**Results:**

Here, a total of 119 *AP2/EREBP* genes were identified in *Fragaria vesca*, and the AP2/EREBP superfamily was divided into AP2, RAV, ERF, DREB, and soloist subfamilies, containing 18, 7, 61, 32, and one member(s), respectively. The DREB subfamily was further divided into six subgroups (A-1 to A-6) based on phylogenetic analysis. Gene structure, conserved motifs, chromosomal location, and synteny analysis were conducted to comprehensively investigate the characteristics of *FvDREBs*. Furthermore, transcriptome analysis revealed distinctive expression patterns among the *FvDREB* genes in strawberry plants exposed to drought stress. The expression of *FvDREB6* of the A-2 subgroup was down-regulated in old leaves and up-regulated in young leaves in response to drought. Furthermore, qRT-PCR analysis found that *FvDREB8* from the A-2 subgroup had the highest expression level under drought stress. Together, analyses with the expression pattern, phylogenetic relationship, motif, and promoter suggest that *FvDREB18* may play a critical role in the regulation of *FvDREB1* and *FvDREB2* expression.

**Conclusions:**

Our findings provide new insights into the characteristics and potential functions of FvDREBs. These *FvDREB* genes should be further studied as they appear to be excellent candidates for drought tolerance improvement of strawberry.

**Supplementary Information:**

The online version contains supplementary material available at 10.1186/s12870-021-03095-2.

## Background

The cultivated strawberry (*Fragaria × ananassa*) is one of the most important and youngest crop species worldwide, originating approximately 300 years ago [11]. Furthermore, strawberry is popular and favored by consumers because of its disease-preventive and medicinal benefits as well as its wide array of aromas and flavors [8, 58]. Genomically, *F. × ananassa* is an allo-octoploid (2n = 8x = 56) plant derived from four different diploid ancestors. Considering the complex genome of *F. × ananassa*, the diploid woodland strawberry (*F. vesca*, 2n = 2x = 14), which has a small completely sequenced genome (240 Mb), is usually used as a model plant for studies on the functions of specific genes and molecular genomic analyses of Rosaceae [49]. A recent report on chromosome-scale assembly in the octoploid strawberry cultivar ‘Camarosa’ has identified a dominant subgenome that was derived from the *F. vesca* progenitor, which largely controls certain metabolic pathways [11]. Strawberry is very sensitive to osmotic stress caused by high-salt or drought stress [44]. Furthermore, strawberry has a large leaf area and a shallow root distribution, and upon drought stress, its growth and yield are greatly affected [15]. Therefore, drought is one of the main limiting factors that affect the growth and quality of strawberry. Understanding the regulatory mechanism of strawberry in response to drought stress can enhance the development and performance of strawberry when subjected to drought stress.

Drought, as a common phenomenon, is expected to intensify with global warming [3, 5]. In plants, a series of molecular, physiological, and biochemical changes caused by the reprogramming of stress-related genes occur in response and adaptation to drought stress [47]. In addition, numerous transcription factors (TFs) have been reported to regulate stress-responsive genes by binding to the promoter region of target genes [2, 6]. The dehydration-responsive element binding (DREB) TFs have been reported to play important roles in response to drought, low-temperature, or high-salt stress [56].

DREB is a subfamily of the APETALA2/ethylene-responsive element binding protein (AP2/EREBP) superfamily, which is categorized into six subgroups (A-1 to A-6) according to the genetic domain [37, 43]. The AP2/EREBPs are unique TFs in plants and characterized by at least one highly conserved AP2 domain. The AP2/EREBPs could be classified as AP2, Related to ABI3/VP1 (RAV), Ethylene Responsive Factor (ERF), DREB, and soloist in *Arabidopsis* and rice [46]. Generally, AP2 subfamily members contain two AP2 domains, RAV subfamily members contain one AP2 domain and an additional B3 domain, and ERF and DREB subfamily members both contain one AP2 domain [40]. The difference between DREB and ERF is based on the association of the AP2 domain with the 14^th^ valine (V14) and 19^th^ glutamic acid (E19) in DREB members, and the 14^th^ alanine (A14) and 19^th^ aspartic acid (D19) in ERF members [46].

With the release of whole-genome sequences for many plant species, more and more AP2/EREBP superfamilies, including DREB subfamilies, have been identified and studied at the genome-wide level [50]. Different DREB subgroups play different roles in different plants. For instance, the overexpression of *AtDREB1A* enhances drought and freezing tolerance in transgenic *Lolium perenne* plants but enhances heat stress tolerance in transgenic chrysanthemum [20, 30], whereas *AtDREB1C* (A-1) plays a central role in stress tolerance in *Arabidopsis* as a negative regulator [42]. Several studies have also reported that the expression of *DREB2A* and *DREB2B*, which belong to the A-2 subgroup, was induced in response to drought stress [31, 45].

Although a recent report had identified 91 *FveERF* genes, the authors had used an old version of the genome and mainly focused on tandem duplications for the expansion of the *FveERF* family [52]. Therefore, there is a lack of information on the expression of *DREBs* in response to drought. An updated annotated version, v4.0.a2, for the *F. vesca* genome has been recently published, adding 9,029 new genes and modifying 8,342 existing genes [32]. In the present study, we identified AP2/EREBP members in the *F. vesca* genome based on the latest version, v4.0.a2, and performed a comprehensive bioinformatics analysis of the DREB subfamily, including DREB classification and naming, gene structure and conserved motif analyses, and chromosomal localization and synteny analyses. Furthermore, the expression profiles of *FvDREBs* in response to drought stress were also obtained using transcriptome and qRT-PCR data. Our results will provide new insights into the biological roles of *FvDREBs*, which may improve drought tolerance in strawberry exposed to drought stress.

## Results

### Identification of AP2/EREBP in *Fragaria vesca*

**Fig. 1:**
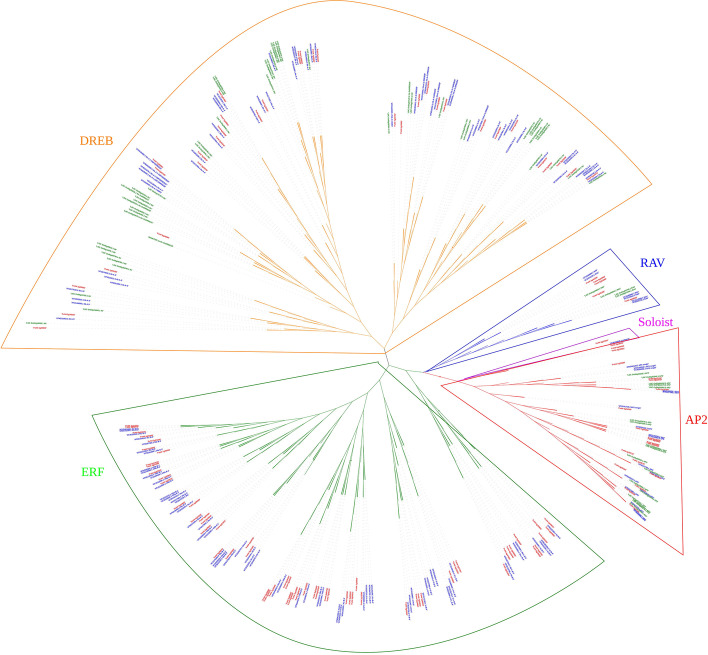
Phylogenetic tree of AP2/EREBP superfamily proteins from *F. vesca*, *Arabidopsis*, and rice. The color of Gene ID of *F. vesca* was red; the color of Gene ID of *Arabidopsis* was blue; the color of Gene ID of rice was green

A total of 119 *AP2/EREBP* genes were identified through HMM searches, local BLAST analyses, and domain confirmations. These genes contained at least one AP2 domain (Supplementary information). A previous study has identified 115 *AP2/EREBP* genes in *F. vesca* [52]*.* The difference between that and this study is that the previous study used an older version of the genome, while our study used the latest version (v4.0.a2). The specific differences in numbers and gene IDs are shown in Table S1. According to the classification of AP2/EREBP in *Arabidopsis* and rice [40, 46], the 119 *AP2/EREBP* genes in *F. vesca* were divided into five groups. A phylogenetic tree was constructed based on the alignment of 337 AP2/EREBP proteins from *Arabidopsis*, rice, and *F. vesca* (Fig. 1; high-resolution Figure 1 in Supplementary material). The phylogenetic tree clearly classified the AP2/EREBP proteins from *F. vesca* into a soloist as well as four typical subfamilies, namely, the AP2, RAV, ERF, and DREB clades, which were comprised of 1, 18, 7, 61, and 32 proteins, respectively. Generally, the RAV subfamily has one AP2 domain and one B3 domain, and the FvRAV subfamily contains two members (FvH4_5g19881 and FvH4_6g29430), which had one AP2 domain and no B3 domain.

### Identification and phylogenetic analysis of *FvDREBs*

Based on the conserved 14^th^ valine (V14) of the AP2 domain (Figure S1), 32 *DREB* genes were identified from AP2/ERF in *F. vesca* and named according to their chromosomal positions (Table 1). The identified FvDREBs proteins ranged from 150 to 579 amino acids in length, with theoretical isoelectric points (pI) ranging from 4.63 to 9.48 and molecular weights (MW) ranging from 16447.59 to 65304.13. Subcellular localization analysis predicted that most (26/32, 81.25%) FvDREBs were localized to the nucleus, whereas other (6/32, 18.75%) members were localized to the cytoplasm (Table 1).

**Table 1:** Features of FvDREBs in *F. vesca*

Gene Name	Gene ID(v1.1)	Gene ID (v4.02)	Chromosome location	Group	No. amino acids	pI	MW	Subcellular localization
*FvDREB1*	mrna32378.1	FvH4_5g01440	Fvb5: 899521-900291	A-1	256	5.20	28817.69	Nucleus
*FvDREB2*	mrna13329.1	FvH4_7g28950	Fvb7: 21384363-21385052	A-1	229	5.13	24962.84	Nucleus
*FvDREB3*	mrna08479.1	FvH4_2g38880	Fvb2: 28047790-28050124	A-2	226	9.48	24777.23	Nucleus
*FvDREB4*	mrna16710.1	FvH4_6g01400	Fvb6: 778696-779238	A-2	180	6.14	19628.61	Nucleus
*FvDREB5*	mrna13783.1	FvH4_6g10690	Fvb6: 6419987-6422452	A-2	579	4.63	65304.13	Cytoplasm
*FvDREB6*	mrna26463.1	FvH4_6g23290	Fvb6: 17276547-17277407	A-2	286	5.78	32216.02	Nucleus
*FvDREB7*	mrna01985.1	FvH4_6g43870	Fvb6: 33904894-33907462	A-2	192	9.40	20839.19	Nucleus
*FvDREB8*	mrna21047.1	FvH4_7g25200	Fvb7: 19265849-19267988	A-2	378	4.89	41645.29	Nucleus
*FvDREB9*	mrna21003.1	FvH4_7g24760	Fvb7: 19064560-19066602	A-3	322	6.30	35598.83	Nucleus
*FvDREB10*	mrna11882.1	FvH4_1g05820	Fvb1: 3086932-3088629	A-4	254	4.95	27529.14	Nucleus
*FvDREB11*	mrna08838.1	FvH4_2g35620	Fvb2: 26279523-26280287	A-4	254	5.01	27654.57	Nucleus
*FvDREB12*	mrna32380.1	FvH4_5g01460	Fvb5: 909587-910426	A-4	279	4.71	29909.66	Nucleus
*FvDREB13*	mrna08876.1	FvH4_5g19440	Fvb5: 11268785-11269904	A-4	225	5.44	25028.56	Nucleus
*FvDREB14*	mrna08873.1	FvH4_5g19460	Fvb5: 11295238-11295834	A-4	187	5.05	20616.61	Nucleus
*FvDREB15*	mrna27021.1	FvH4_5g33220	Fvb5: 24034746-24036098	A-4	196	5.26	21569.16	Nucleus
*FvDREB16*	mrna27017.1	FvH4_5g33240	Fvb5: 24050928-24051485	A-4	185	4.97	20338.81	Nucleus
*FvDREB17*	mrna30159.1	FvH4_6g18000	Fvb6: 11817696-11821178	A-4	239	6.43	25517.71	Cytoplasm
*FvDREB18*	mrna30226.1	FvH4_6g18090	Fvb6: 11874334-11874957	A-4	207	5.02	22976.03	Cytoplasm
*FvDREB19*	mrna25758.1	FvH4_6g32030	Fvb6: 25127142-25128659	A-4	258	5.03	27960.85	Nucleus
*FvDREB20*	mrna04810.1	FvH4_7g09550	Fvb7: 9175646-9176470	A-4	274	5.24	29760.18	Nucleus
*FvDREB21*	mrna19141.1	FvH4_7g16810	Fvb7: 14364300-14365229	A-4	198	5.43	21984.29	Nucleus
*FvDREB22*	mrna13327.1	FvH4_7g28960	Fvb7: 21396222-21397656	A-4	235	4.92	24643.19	Nucleus
*FvDREB23*	mrna12919.1	FvH4_1g09180	Fvb1: 4892045-4893214	A-5	216	4.69	24040.38	Nucleus
*FvDREB24*	mrna23873.1	FvH4_1g16370	Fvb1: 9445234-9446402	A-5	150	9.45	16447.59	Cytoplasm
*FvDREB25*	mrna11145.1	FvH4_2g26630	Fvb2: 21353932-21356192	A-5	234	5.37	25569.60	Nucleus
*FvDREB26*	mrna09137.1	FvH4_2g34020	Fvb2: 25362442-25364608	A-5	159	9.18	18020.95	Nucleus
*FvDREB27*	mrna27062.1	FvH4_5g33180	Fvb5: 23967454-23968327	A-5	165	5.50	17698.44	Cytoplasm
*FvDREB28*	mrna26530.1	FvH4_5g34550	Fvb5: 25211129-25211910	A-5	209	7.62	23054.50	Cytoplasm
*FvDREB29*	mrna16350.1	FvH4_1g21210	Fvb1: 13220000-13221070	A-6	356	6.00	39556.12	Nucleus
*FvDREB30*	mrna32084.1	FvH4_5g04470	Fvb5: 2609265-2610158	A-6	297	8.55	32973.25	Nucleus
*FvDREB31*	mrna22114.1	FvH4_5g37820	Fvb5: 27851089-27852207	A-6	372	5.85	40638.05	Nucleus
*FvDREB32*	mrna17698.1	FvH4_6g26090	Fvb6: 19953052-19954410	A-6	452	5.76	51223.92	Nucleus

**Fig. 2:**
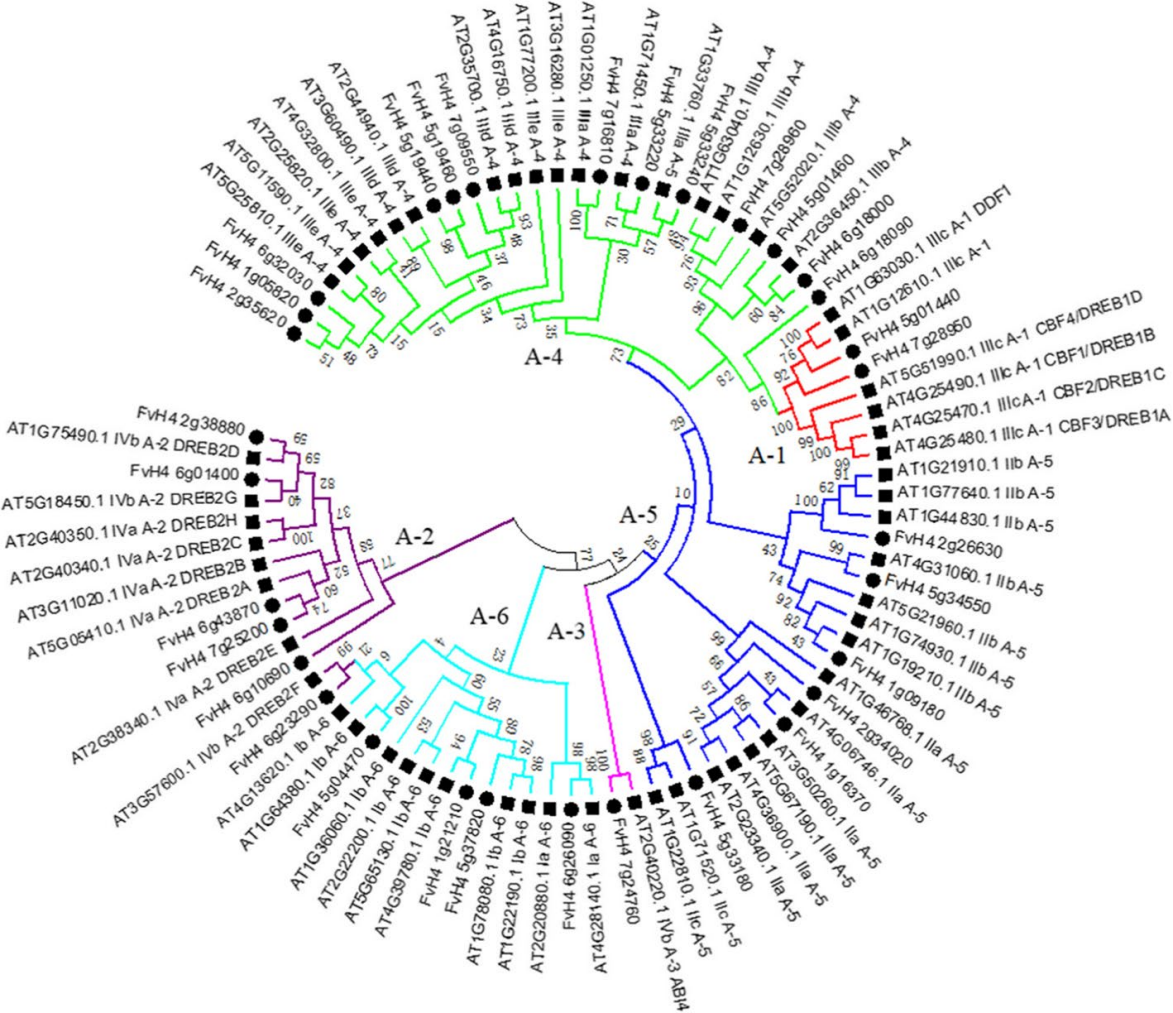
Phylogenetic tree of DREB subfamily proteins from *F. vesca* and *Arabidopsis*. Different subgroups (A-1 to A-6) of DREB subfamily proteins are highlighted in different colors

To investigate the phylogenetic relationships between DREBs in strawberry and other plants, a neighbor-joining phylogenetic tree was generated using the whole-protein sequences of the DREB subfamily between *F. vesca* and *A. thaliana*. As shown in Fig. 2, the phylogenetic tree was further divided into six subgroups (A-1 to A-6) as in *Arabidopsis*, in which the A-4 subgroup was the largest (13 members) and the A-3 subgroup was the smallest (one member). According to the similarities between AtDREB1/CBF and AtDREB2, the A-1 subgroup and A-2 subgroup included two and six members, respectively. Meanwhile, seven orthologous pairs were identified in *F. vesca* and *A. thaliana*, and one paralogous pair was identified in *F. vesca* based on a bootstrap value greater than 90 (Supplementary information).

### Gene structure and conserved motif analysis of FvDREBs

**Fig. 3:**
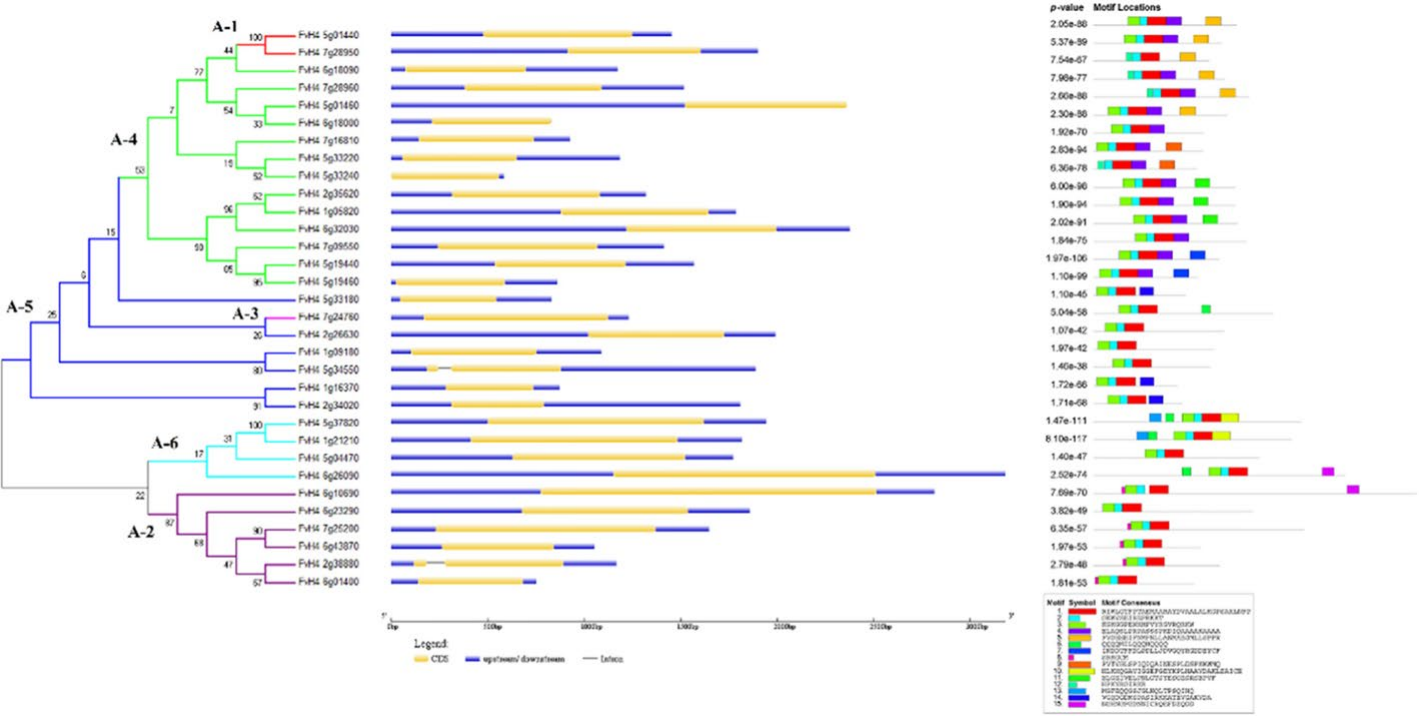
Phylogenetic relationships, exon/intron structures, and conserved motifs of FvDREBs in *F. vesca*. Different subgroups (A-1 to A-6) are highlighted in different color. Different motifs are represented by blocks of different color and size

The exon-intron structures were analyzed to gain a better understanding of the structural characteristics of the *FvDREBs* genes. Almost all FvDREBs (30/32, 93.75%) were intronless, except for FvH4_2g38880 (FvDREB3) and FvH4_5g34550 (FvDREB28), which contained only one intron (Fig. 3).

The conserved motifs of all FvDREBs were further examined using MEME. A total of 15 motifs were predicted and named as motifs 1 to 15. Motifs 1 and 2 were found in all FvDREB protein sequences and were related to the AP2 domain. The protein sequences of two members belonging to the A-1 subgroup both contained motifs 5 and 11. Motif 8 was only found in members of the A-2 subgroup, whereas motif 10 was only found in members of the A-6 subgroup. Some other motifs, such as motif 15, were distributed among various subgroups.

### Chromosomal location and tandem duplication of FvDREBs

**Fig. 4:**
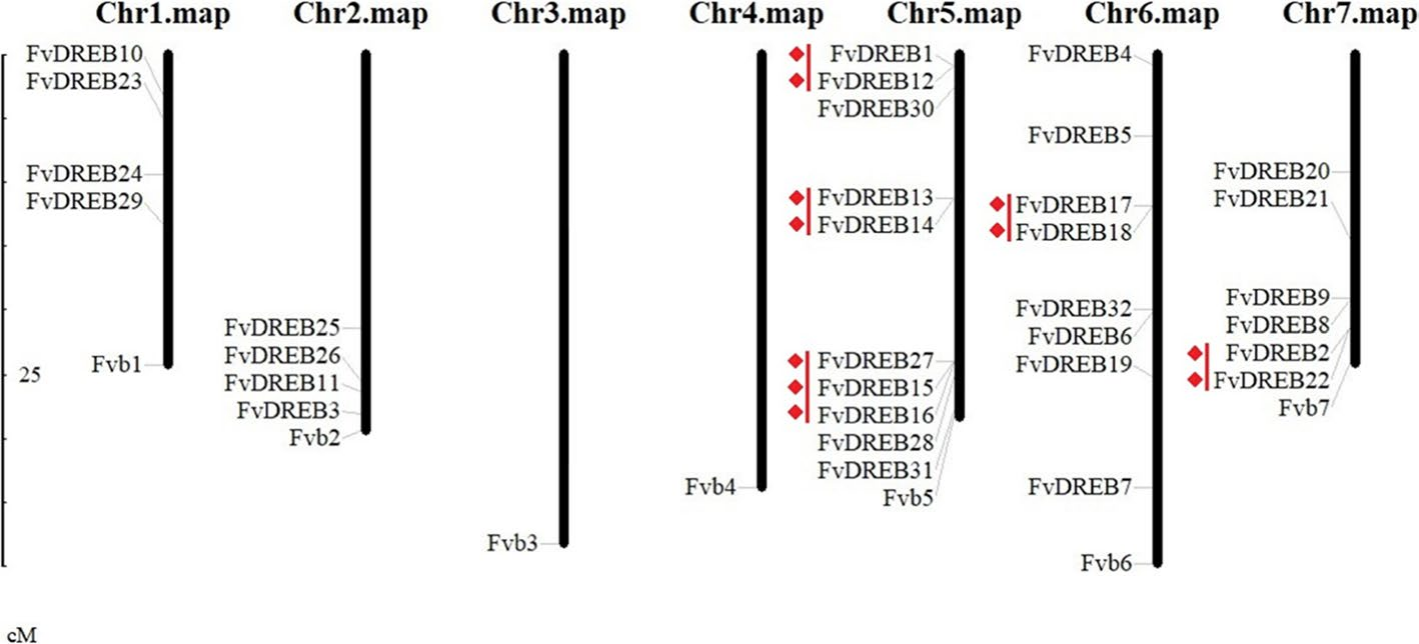
Chromosomal locations of *FvDREBs* in *F. vesca*. Red lines indicate tandem duplications

In order to explore the functional differentiation of FvDREB members, their positions on chromosomes were further investigated with the latest annotated genome (v4.0.a2). As shown in Fig. 4, 32 FvDREB members were distributed unevenly on five of the seven chromosomes, and there were no members on chromosomes 3 and 4. Chromosome 5 had the largest number (10, 31.25%) of *FvDREB* genes, containing one A-1 subgroup member, five A-4 subgroup members, two A-5 subgroup members, and two A-6 subgroup members. Chromosomes 6 and 7 had 25% (8/32) and 18.75% (6/32) *FvDREB* genes, respectively. The remaining 25% (8/32) members were evenly distributed on chromosomes 1 and 2. Moreover, five tandem duplication events involving eleven *FvDREB* genes were observed, namely, *FvDREB1* and *FvDREB12*, *FvDREB13* and *FvDREB14*, *FvDREB17* and *FvDREB18*, *FvDREB2* and *FvDREB22*, and *FvDREB27*, *FvDREB15,* and *FvDREB16*. Three of the five tandem duplication events were distributed on chromosome 5, including the three members with tandem duplications.

### Synteny analysis of FvDREBs

**Fig. 5:**
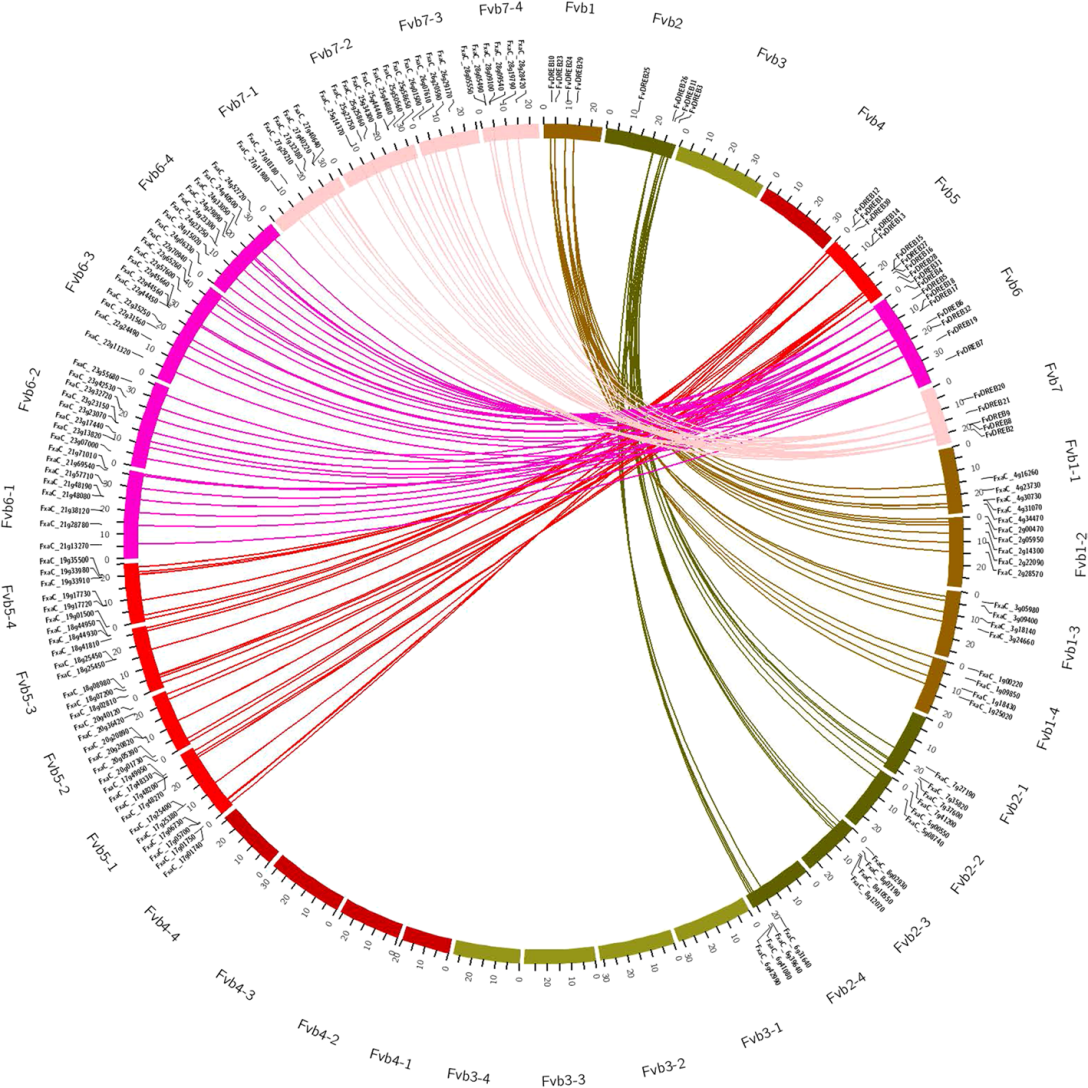
Syntenic relationships of DREB members from *F. vesca* and *F. × ananassa*

There were 143 syntenic gene pairs that were syntenic between *F. vesca* and *F. × ananassa*, and high levels of collinearity were observed in all *FvDREBs* between *F. vesca* and their corresponding *F. × ananassa*, except *FvDREB24* (Fig. 5). Moreover, each FvDREBs in *F. vesca* chromosomes corresponded to several syntenic genes in *F. × ananassa* chromosomes. For example, the *FvDREB10* gene was on chromosome 1 in *F. vesca* and its syntenic corresponding genes were on chromosome Fvb1-1, Fvb1-2, Fvb1-3, and Fvb1-4, respectively, in *F. × ananassa*.

### Expression profiles of FvDREBs in response to drought stress in different strawberry leaves

**Fig. 6:**
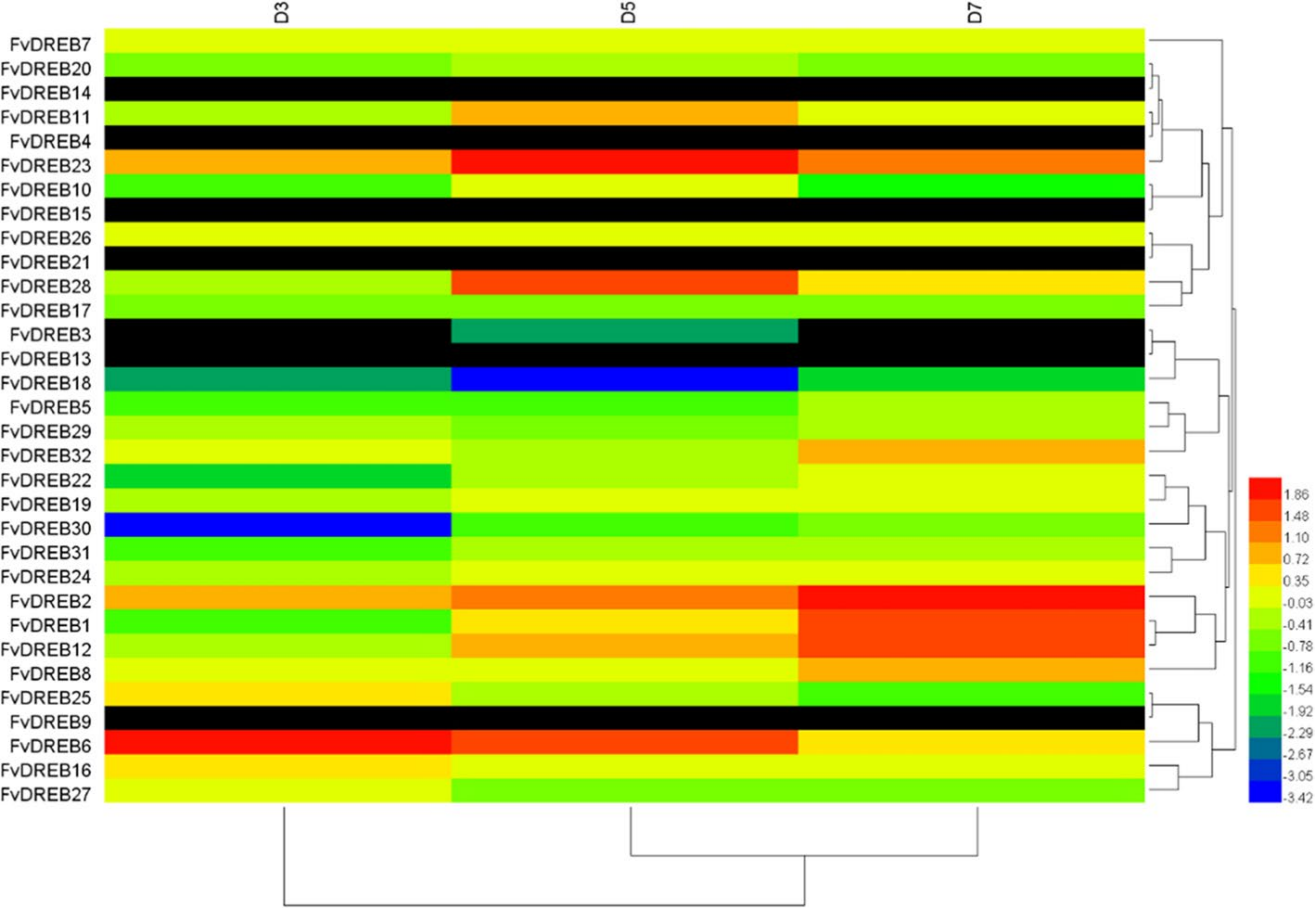
Heat map of differentially expressed *FvDREBs* in strawberry old leaves under drought stress. Red indicates up-regulation, blue and green indicate down-regulation, and black indicates data gaps

**Fig. 7:**
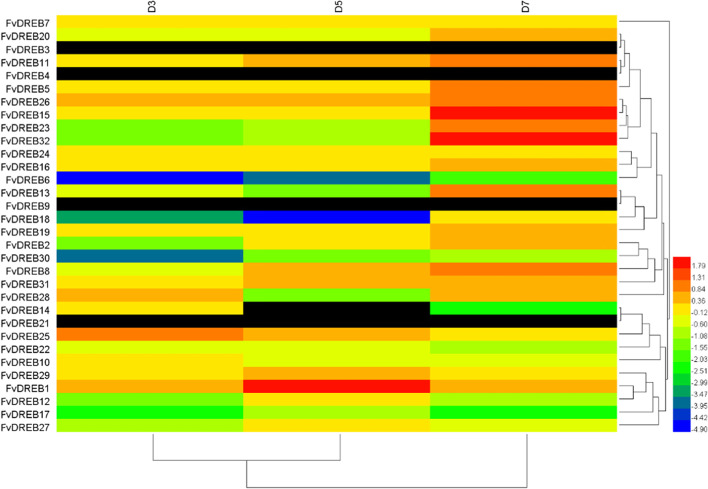
Heat map of differentially expressed *FvDREBs* in strawberry young leaves under drought stress. Red indicates up-regulation, blue and green indicate down-regulation, and black indicates data gaps

Transcriptome sequencing data from old and young leaves exposed to different drought stress conditions were previously generated by our group to investigate the expression profiles of *FvDREBs*. In general, different subgroups from different tissues showed different expression patterns (Figs. 6 and 7), suggesting functional divergence between different subgroups of *FvDREB* members. In old leaves, D5 and D7 showed a similar clustering relationship (Fig. 6), whereas D3 and D5 displayed a similar clustering relationship in young leaves (Fig. 7). Two genes (*FvDREB1* and *FvDREB2*) from the A-1 subgroup were highly expressed in the later period of drought stress, whereas the expression of *FvDREB6*, which belonged to the A-2 subgroup, was significantly up-regulated at the initial stage of drought stress. The expression of *FvDREB30* from the A-6 subgroup was lower in the early stages of drought stress and that of *FvDREB18* from the A-4 subgroup was lower in the middle stages of drought stress. Their expression levels in old and young leaves were similar (Figs. 6 and 7), indicating that they are negative regulators in response to drought stress.

**Fig. 8:**
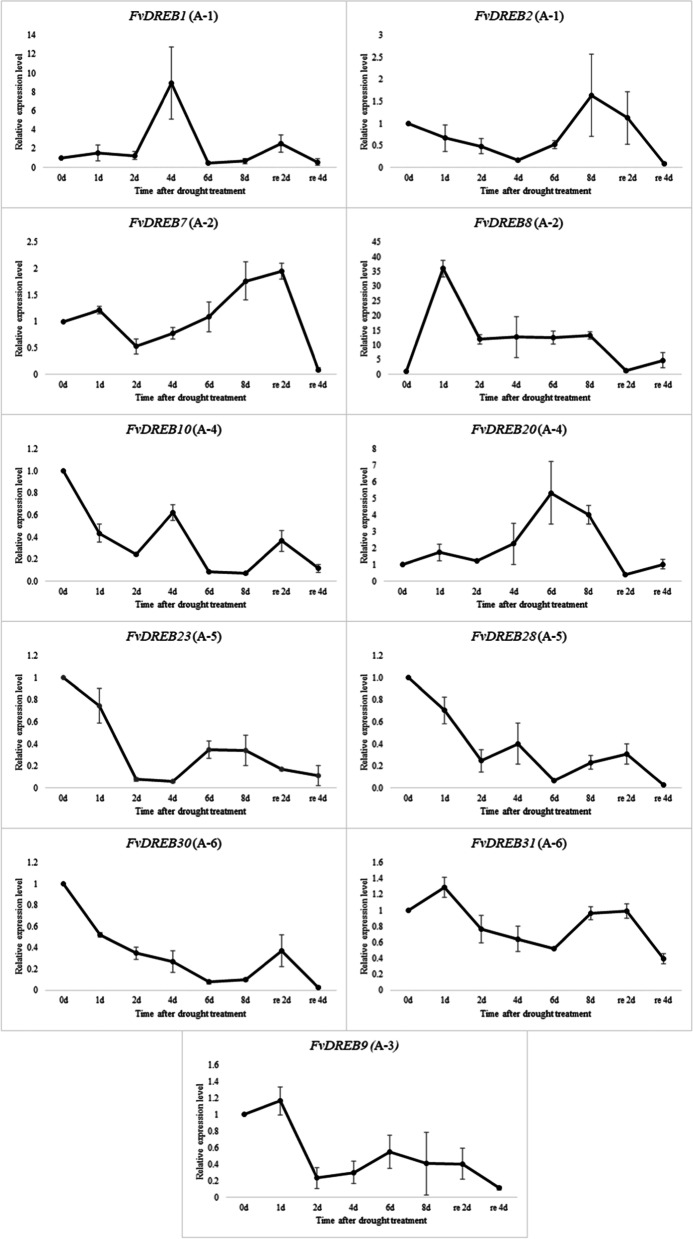
Expression profiles of the *FvDREB* genes in response to drought stress

To further verify the expression of these identified *FvDREB* genes, two genes were randomly selected from each subgroup of the *FvDREB* gene family (the A-3 subgroup had only one member, so only one gene was selected) to detect their expression levels under different drought stress conditions by qRT-PCR analysis (Fig. 8). The results showed that the expression of *FvDREB8* of the A-2 subgroup was significantly up-regulated, with the highest expression observed at 1d under drought stress (Fig. 8). The expression level gradually decreased, but the expression levels of all genes were ten times higher than those in the control group. The expression level of *FvDREB1* from the A-1 subgroup reached the highest level when it was subjected to drought stress for 4 days, which was more than eight times that of the control group (Fig. 8). The expression level of *FvDREB20* from the A-4 subgroup reached the highest level when subjected to drought stress for 6 days, which was more than five times that of the control group (Fig. 8). The longer the time of exposure to drought stress, the greater the down-regulation of *FvDREB30* from the A-6 subgroup. The trend of the expression of *FvDREB* was consistent with the RNA-Seq data. It could be seen that the expression of *FvDREB* genes from different subgroups was variable and unstable under drought stress.

## Discussion

AP2/EREBP TFs are one of the largest families of plant-specific transcriptional regulators that modulate many plant processes such as plant growth, development, and response to various stresses. The AP2/EREBP superfamily has been widely identified and investigated in diverse plant species with the release of the whole-genome sequences of various plants. Furthermore, continuous improvements in high-throughput sequencing techniques and bioinformatic algorithms have allowed the accurate and accelerated delivery of information for researchers [1]. In this study, we identified the AP2/EREBP superfamily and comprehensively analyzed the DREB subfamily in the *F. vesca* genome based on the latest version, v4.0.a2. Moreover, we used RNA-Seq (transcriptome sequencing) and qRT-PCR to characterize differentially expressed *FvDREB* genes when plants were exposed to drought stress. Our study provides a better understanding of the FvDREB proteins, which may benefit strawberry biotechnological breeding and improve their adaptation and tolerance to drought.

The DREB TF family plays important roles in the response to abiotic stress. However, knowledge of *FvDREB* genes is limited. Gene structure analysis of *FvDREBs* revealed that 93.75% of *FvDREBs* were intronless, which was higher than that of rice, maize, and other plants [24, 27]. Motif analysis showed that all FvDREB protein sequences had motifs related to the AP2 domain and demonstrated that the AP2 domain was highly conserved in FvDREBs. The results also showed that different subgroups had specific motifs (Fig. 3). Generally, the DREB sequences were confirmed based on the presence of an AP2 domain with the 14^th^ valine (V14) and 19^th^ glutamic acid (E19) [46]. A comparison of the amino acid sequences of AP2 domains in FvDREBs revealed that although all of the amino acid sequences at position 14 were valine (V14), those at position 19 were not all glutamic acid (E19) (Figure S1). In addition, 81.25% of FvDREB proteins were predicted to be localized to the nucleus, suggesting the roles of these proteins in signal transduction and transcriptional regulation [48]. These results indicate that most domains or motifs of FvDREB proteins were highly conserved and functional divergence may occur by changing key amino acids. ERFs and DREBs exhibit distinctive features, the ERFs are bound to the GCC box (AGCCGCC), whereas the DREBs are bound to DRE (A/GCCGAC) [34].

Whole-genome duplications (WGD), tandem duplications, segmental duplications, or polyploidization events are widely believed to be the primary sources of plant evolution, they have also contributed to gene family expansion [4, 19, 25]. All FvDREB proteins were distributed unevenly on five of the seven chromosomes, which might have been the result of WGD. Tandem duplications and segmental duplications were also detected in FvDREBs, which also contributed to the expansion of the *FvDREB* gene family. Furthermore, we also performed an intergenomic synteny analysis between *F. vesca* and *F. × ananassa* to study the evolution of FvDREB in the *Fragaria* genome along with the species evolution and polyploidization. The high level of collinearity between FvDREBs of diploid and their corresponding octoploid homologs suggests a close relationship between genomes in *Fragaria* [11].

The expression patterns of FvDREBs reflect biological roles and gene functions in response to drought stress. Interestingly, we found that the expression of *FvDREB1* from the A-1 subgroup correlated with the early and middle stages of drought stress, whereas the expression of *FvDREB2* from the A-1 subgroup correlated with the middle and late stages of drought stress (Fig. 8). This suggests that the individual contribution and function of each DREB are different in response to the entire drought stress process. The A-1 of DREBs, also known as C-repeat binding factors (CBFs), were first identified as TFs in response to both low temperature and osmotic stress in *Arabidopsis* [56]. Furthermore, more and more studies have demonstrated that the overexpression of *DREB1/CBF* can improve the tolerance to environmental stresses such as freezing, drought, salt, and high temperature [16, 18, 23]. Our previous meta-analysis of the effect of *CBF/DREB* overexpression on drought stress response also confirms the premise that *DREB* overexpression can enhance drought stress tolerance in various crops and reflect the duration of the stress treatment (stress time). As a moderator, it had a clear effect on the response of the transgenic plants in relation to some parameters [10].

Equally important, the fact that *FvDREB18* was down-regulated in response to whole drought stress, especially in the middle stage of drought stress of both old and young leaves, combined with the expression pattern of *FvDREB1* and *FvDREB2*, was very similar to that of *CBF2/DREB1C*, a negative regulator of *CBF1/DREB1B* and *CBF3/DREB1A* in *Arabidopsis* [42]. The clade containing *FvDREB18* was closest to A-1 subgroup clades in the phylogenetic tree with a bootstrap value of 86 (Fig. 2), and the number of conserved motifs in *FvDREB18* was four, whereas the number of conserved motifs in *FvDREB1* and *FvDREB2* was five (Fig. 3). This may suggest that *FvDREB18* plays a critical role in response to drought stress by precisely controlling the expression of *FvDREB1* and *FvDREB2*, and, hence, that of the downstream genes. Moreover, we analyzed the promoters of these three genes and found that there were different cis-regulatory elements among them (Supplementary information and Figure S2). The cis-regulatory elements, such as ABRE (cis-acting element involved in the abscisic acid responsiveness), ARE (cis-acting regulatory element essential for the anaerobic induction), MBS (MYB binding site involved in drought-inducibility), MYB, and MYC, were present in the promoter regions of all three genes. By contrast, CAT-box, TGA-element, and HD-Zip 1 were only detected in the *FvDREB2* promoter, which was related to meristem expression, auxin-responsive, and the differentiation of palisade mesophyll cells, respectively. There were three TCA-element elements (cis-acting element involved in salicylic acid responsiveness) in the *FvDREB2* promoter and one in the *FvDREB1* promoter, but none in the *FvDREB18* promoter. The different types and numbers of cis-regulatory elements play essential roles in determining the stress-responsive or tissue-specific expression patterns of genes [13, 35], and those presenting in the promoter region of *FvDREBs* may indicate differential regulatory networks. However, the mechanisms by which *FvDREB18* regulates *FvDREB1* and *FvDREB2* expression and the involvement of other regulators await further investigation.

In addition, *FvDREB8* from the A-2 subgroup was significantly and positively regulated by drought stress and induced strongly and rapidly in the early stage. This suggests that *FvDREB8* may be very sensitive to drought. The A-2 subgroup DREB members, which are involved in drought-responsive gene expression, were first referred to as DREB2 to distinguish them from DREB1, whereas DREB1 is thought to function in cold-responsive gene expression regulation [47]. DREB2A and DREB2B are induced under drought and salt stress conditions as two of the total eight DREB2-type genes in *Arabidopsis*, and only *OsDREB2A* and *OsDREB2B* were found to be induced by abiotic stress as two of all five DREB2-type genes in rice [41, 39, 46]. In this study, in addition to *FvDREB8*, *FvDREB6* also showed drought stress-inducible gene expression among all six A-2 subgroup genes in *F. vesca*. The expression of *FvDREB6* was down-regulated in old leaves and up-regulated in young leaves in response to drought and appeared to be tissue-specific (Figs. 6 and 7). Remarkably, the gene annotated as *DREB2-2* was down-regulated in the leaves of dehydrated *B. napus*, whereas its expression was increased in roots [22, 33]. These findings provide new insights into the genetic control of drought tolerance in strawberry and offer some useful candidates for drought tolerance improvement.

## Conclusions

We performed a genome-wide analysis on the AP2/EREBP family genes in *F. vesca* and identified 119 *FvAP2/EREBP* genes. A detailed investigation of the classification, phylogenetic evolution, structure, synteny, and expression profile of these FvDREBs in different tissues and in response to drought stress was carried out. Our results reveal that *FvDREB8* from the A-2 subgroup play crucial roles in the early stage of drought stress response. *FvDREB6* appeared to be tissue-specific and *FvDREB18* may play a critical role in regulating the expression of *FvDREB1* and *FvDREB2.* Overall, our findings provide new insights into the characteristics and potential functions of FvDREBs and offer a better understanding of their molecular basis in response to drought stress in strawberry.

## Methods

### Identification and classification of the *DREB* genes in strawberry

The most recent version of the *F. vesca* genome v4.0.a2 was downloaded from the Genome Database for Rosaceae (GDR) (https://www.rosaceae.org/species/fragaria_vesca/genome_v4.0.a2) to identify strawberry DREB TFs. Additionally, 53 *DREB*, 23 *AP2*, and six *RAV* genes from rice (*Oryza sativa*) were downloaded from the MSU Rice Genome Annotation Project Database (RGAP) (http://rice.plantbiology.msu.edu//), and 148 *AP2/EREBP* genes from *Arabidopsis* were downloaded from The *Arabidopsis* Information Resource (TAIR) (https://www.arabidopsis.org/index.jsp) database. The Hidden Markov Model (HMM) of the AP2 domain (PF00847) was downloaded from the Pfam protein analysis website (http://pfam.xfam.org/) and used to identify AP2/EREBPs with a defined e-value threshold < 1e−5. To search for all possible AP2/EREBPs, some AtDREB members were used as the query sequence in the local Basic Local Alignment Search Tool (BLAST). To validate the search results, all candidate sequences were examined and analyzed by a simple modular architecture research tool (SMART) (http://smart.embl.de/) [29] and the Conserved Domain Database (CDD) (http://www.ncbi.nlm.nih.gov/Structure/cdd/wrpsb.cgi) [38].

All the AP2/EREBP candidate sequences encoding the conserved AP2 domain were constructed with multiple alignments by MUSCLE [12]. A neighbor-joining phylogenetic tree was generated using the 1000 bootstrap method and Poisson model with MEGA 6.06 [51]. All AP2/EREBP proteins were aligned to *Arabidopsis* and rice AP2/EREBP proteins to classify them into different groups. And the DREBs were identified based on the presence of only one AP2 domain with the 14^th^ valine (V14) and 19^th^ glutamic acid (E19) [40, 46]. The theoretical isoelectric point (pI) and molecular weight (MW) of the identified proteins were analyzed by the ProtParam Tool (https://web.expasy.org/protparam/) [14]. The subcellular localization of each protein was predicted with Cell-PLoc 2.0 (http://www.csbio.sjtu.edu.cn/bioinf/Cell-PLoc-2/) [7].

### Gene structure and conserved motif analysis of the *FvDREB* genes

The coding sequences (CDS) and full-length sequences of *FvDREB* genes were obtained from NCBI and graphically displayed with Gene Structure Display Server 2.0 (GSDS) (http://gsds.cbi.pku.edu.cn/) [21]. Conserved motifs in *FvDREBs* were predicted by the Multiple Em for Motif Elicitation Tool 5.1.1 (MEME) (http://meme-suite.org/tools/meme) using default parameters.

### Chromosomal localization and synteny analyses

The chromosomal locations of the *FvDREBs* were retrieved from the annotated file of the *F. vesca* genome v4.0.a2 and graphically represented with MapInspect (http://www.softsea.com/review/MapInspect.htm). To investigate the effect of genome duplications on DREB evolution, we conducted an intergenomic synteny analysis on *F. vesca* and *F. × ananassa*. The syntenic information of *FvDREBs* was calculated with MCScanX [54], and the syntenic diagram was visualized with Circos (http://circos.ca/) [26]. Tandem duplications were characterized as multiple members of *FvDREBs* occurring in neighboring intergenic regions (distance < 100 kb) that were separated by ten or fewer non-homologous spacer genes [19]. Segmental duplications were identified from the Plant Genome Duplication Database (PGDD) (http://chibba.agtec.uga.edu/duplication/) [28].

### Plant materials and stress treatment

The strawberry (*F. vesca* subspecies vesca) seeds were a kind gift from Dr. Janet Slovin (Fruit and Vegetable Lab at the USDA). They were grown on 1/2 MS medium after disinfecting with NaClO (20%, 20 mins) in a tissue culture room for 6 weeks with a photoperiod of 16-h light/8-h dark and a temperature of 24 ± 2°C. Light (~200 μmol m^−2^ s^−1^) was supplied by LED.

Drought stress was applied by transferring plants to soil medium and then withholding water. All leaves were collected from each 2-month-old plant which exposed to drought stress at 0 d, 1 d, 2 d, 4 d, 6 d and 8 d, as well as 2 d, 4 d post treatment. All samples were immediately placed into liquid nitrogen and stored at -80°C. Three biological replicates were analyzed for each treatment and control.

Total RNA was extracted from strawberry leaves using the CTAB method with minor modifications [17]. The RNase-free DNase Set with RNeasy/QIAamp® Columns (QIAGEN, USA) was used to eliminate contaminating genomic DNA. Total RNA was reverse transcribed into cDNA using the High Capacity cDNA Reverse Transcription Kit (Applied Biosystems, USA). All cDNAs were stored at -20°C.

### Transcriptome analysis of strawberry under drought stress

The transcriptome data, which were unpublished, were provided by a member of our lab, Xiaolong Wang, and were only analyzed in his doctoral dissertation [53]. The strawberries were exposed to drought stress after 1 day of full water absorption. D3, D5, and D7 samples were collected on the third day (after 2 days of drought treatment), fifth day (after 4 days of drought treatment), and seventh day (after 6 days of drought treatment), respectively. Transcriptome data were obtained from a fixed amount of RNA collected from old (the stage of fully expanded mature leaves) and new leaves (the stage before fully expanded mature leaves). The log2 transformed Fragments per Kilobase per Million mapped reads (Log^2^FPKM) was used to calculate the expression levels of genes in control and treated plants at different times. The different expression patterns of *FvDREB* genes (Supplementary information) were clustered and visualized by HemI (http://hemi.biocuckoo.org/down.php) [9].

### qRT-PCR and expression pattern analysis

The expression patterns of *FvDREB* genes were examined by quantitative real-time Polymerase Chain Reaction (qRT-PCR) using the QuantStudio^TM^ Flex 96-Well PCR System (Applied Biosystems, USA) and SYBR® Green Reagents (Applied Biosystems, USA). The primer sequences used are listed in the Supplementary information. Some primers were designed with qPrimerDB (http://biodb.swu.edu.cn/qprimerdb), and others were designed with Beacon Designer 8.14. The total volume of each reaction mixture was 10 μL; it included 1 μL of cDNA as the template, 5 μL of PowerUp^TM^ SYBR® Green Master Mix (Applied Biosystems, USA), 0.6 μL of each primer, and 3.4 μL of ddH_2_O. The PCR cycling conditions were as follows: 95°C for 10 min, followed by 40 cycles of amplification for 30 s at 95°C, annealing for 30 s at 58–60°C (depending on the primer’s annealing temperature), and extension for 20 s at 72 °C. The melting curve conditions were as follows: 72°C to 95°C for 15 s, 60°C for 1 min, and 95°C for 15 s. This was performed for each amplification immediately after the PCR. Four commonly used reference genes (*actin*, *EF1*, *GAPDH*, *DBP*) were amplified to test the stability of the expressed genes in strawberry. Finally, *EF1* was used in this study because it was the most stable of the four reference genes.

The relative expression levels were calculated using the 2^-ΔΔCT^ (ΔCT = CT target – CT reference; ΔΔCT = (CT target – CT reference) treatment – (CT target – CT reference) control) method [36]. The standard deviation (SD) were calculated by the three biological replicates [57].

## Supplementary Information

**Additional file 1.** The details information of AP2/EPEBP and DREB contain gene name, gene ID, homologous, synteny, segmental duplication, promoter analysis, primer sequence and transcriptome data.

**Additional file 2: Table S1**. Comparison of group/subgroup size of AP2/EREBP superfamily between this study and the previous study. **Figure S1**. Comparison of amino acid sequences of the AP2 domains in the FvDREB subfamily. **Figure S2**. Characteristics of *cis*-regulatory elements in the promoter region of *FvDREBs*.

## Data Availability

All data generated or analyzed during this study are included in this published article and its supplementary information files. The transcriptomic data supporting the conclusions of this article come from the doctoral dissertation of Xiaolong Wang [53], and the accession number of this transcriptomic data is PRJNA733854.

## Abbreviations

AP2/EREBP: APETALA2/ethylene-responsive element binding protein
BLAST: Basic Local Alignment Search Tool
CBFs: C-repeat binding factors
CDD: Conserved Domain Database
DREB: Dehydration-responsive element binding protein
ERF: Ethylene Responsive Factor
*F. × ananassa*: *Fragaria × ananassa*
*F. vesca*: *Fragaria vesca*
GDR: Genome Database for Rosaceae
GSDS: Gene Structure Display Server
HMM: Hidden Markov Model
MEME: Multiple Em for Motif Elicitation Tool
MW: Molecular weight
PGDD: Plant Genome Duplication Database
pI: Isoelectric point
qRT-PCR: Quantitative real-time Polymerase Chain Reaction
RAV: Related to ABI3/VP1
RGAP: MSU Rice Genome Annotation Project Database
TAIR: The *Arabidopsis* Information Resource
TFs: Transcription factors
WGD: Whole-genome duplication
