# Supplementary material for: Genome-wide identification of AP2/EREBP in Fragaria vesca and expression pattern analysis of the FvDREB subfamily under drought stress
Source: BMC Plant Biol. 2021 Jun 26;21:295. doi: 10.1186/s12870-021-03095-2 (PMC8236174; doi:10.1186/s12870-021-03095-2)
Supplement: Supplementary file 2 — Additional file 2: Table S1. Comparison of group/subgroup size of AP2/EREBP superfamily between this study and the previous study. Figure S1. Comparison of amino acid sequences of the AP2 domains in the FvDREB subfamily. Figure S2. Characteristics of cis-regulatory elements in the promoter region of FvDREBs. [file 12870_2021_3095_MOESM2_ESM.docx]

Table S1. Comparison of group/subgroup size of AP2/EREBP superfamily between this study and the previous study.

| This study | | | Previous study (Wang et al., 2019) | | |
| --- | --- | --- | --- | --- | --- |
| Classification | Group | No. | Classification | Group | No. |
| AP2 family |  | 18 | AP2 family |  | 18 |
|  | Double AP2 domain | 16 |  |  |  |
|  | Single AP2 domain | 2 |  |  |  |
| AP2/ERF family |  | 93 | ERF family |  | 91 |
|  | DREB | 32 |  | Group 1-5 | 33 |
|  | ERF | 61 |  | Group 6-11 | 58 |
|  |  |  |  |  |  |
| Soloist |  | 1 | Soloist |  | 1 |
| RAV family | RAV | 7 | RAV family |  | 5 |
|  |  |  |  |  |  |
|  | Total | 119 |  | Total | 115 |


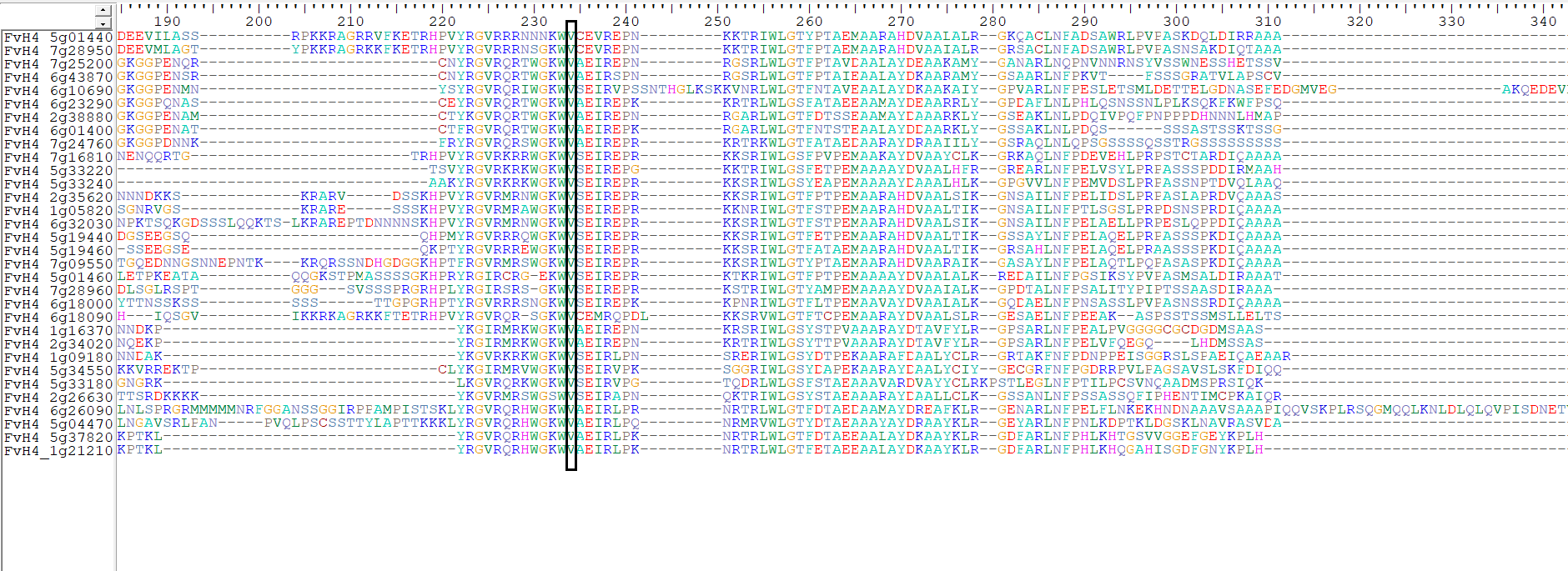


Fig. S1. Comparison of amino acid sequences of the AP2 domains in the FvDREB subfamily


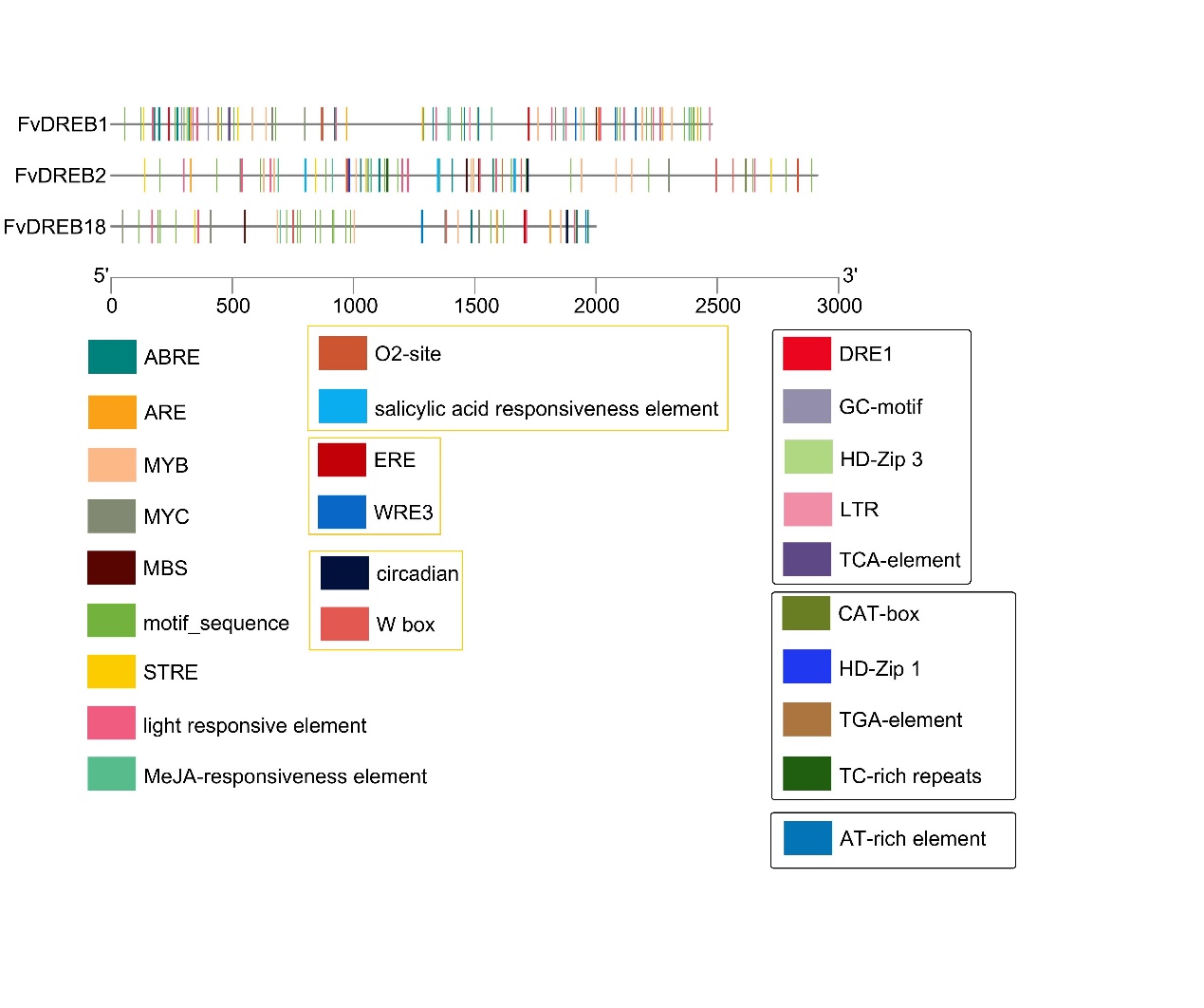


Fig. S2. Characteristics of cis-regulatory elements in the promoter region of *FvDREBs*. All cis-regulatory elements were listed in supplementary information. Some basic cis-acting elements, such as TATA-box, GC-BOX and CAAT-box, and some cis-regulatory elements with unknown functions were not shown in this figure. The elements in the left column were found in the promoter region of the three *FvDREB* genes, in the middle column were found in the two of three genes, and in the right column were only found in one of the three gene.
